# Gene Polymorphisms in Boar Spermatozoa and Their Associations with Post-Thaw Semen Quality

**DOI:** 10.3390/ijms21051902

**Published:** 2020-03-10

**Authors:** Anna Mańkowska, Paweł Brym, Łukasz Paukszto, Jan P. Jastrzębski, Leyland Fraser

**Affiliations:** 1 Department of Animal Biochemistry and Biotechnology, Faculty of Animal Bioengineering, University of Warmia and Mazury in Olsztyn, 10-719 Olsztyn, Poland; anna.mankowska@uwm.edu.pl; 2 Department of Animal Genetics, Faculty of Animal Bioengineering, University of Warmia and Mazury in Olsztyn, 10-719 Olsztyn, Poland; pawbrym@uwm.edu.pl; 3 Department of Plant Physiology and Biotechnology, Faculty of Biology and Biotechnology, University of Warmia and Mazury in Olsztyn, 10-719 Olsztyn, Poland; pauk24@gmail.com (Ł.P.); bioinformatyka@gmail.com (J.P.J.)

**Keywords:** freezability, RNA-Seq, variant calling, genotype, SNPs

## Abstract

Genetic markers have been used to assess the freezability of semen. With the advancement in molecular genetic techniques, it is possible to assess the relationships between sperm functions and gene polymorphisms. In this study, variant calling analysis of RNA-Seq datasets was used to identify single nucleotide polymorphisms (SNPs) in boar spermatozoa and to explore the associations between SNPs and post-thaw semen quality. Assessment of post-thaw sperm quality characteristics showed that 21 boars were considered as having good semen freezability (GSF), while 19 boars were classified as having poor semen freezability (PSF). Variant calling demonstrated that most of the polymorphisms (67%) detected in boar spermatozoa were at the 3’-untranslated regions (3’-UTRs). Analysis of SNP abundance in various functional gene categories showed that gene ontology (GO) terms were related to response to stress, motility, metabolism, reproduction, and embryo development. Genomic DNA was isolated from sperm samples of 40 boars. Forty SNPs were selected and genotyped, and several SNPs were significantly associated with motility and membrane integrity of frozen-thawed (FT) spermatozoa. Polymorphism in *SCLT1* gene was associated with significantly higher motility and plasma membrane integrity of FT spermatozoa from boars of the GSF group compared with those of the PSF group. Likewise, polymorphisms in *MAP3K20, MS4A2,* and *ROBO1* genes were significantly associated with reduced cryo-induced lipid peroxidation and DNA damage of FT spermatozoa from boars of the GSF group. Candidate genes with significant SNP associations, including *APPL1, PLBD1, FBXO16, EML5, RAB3C*, *OXSR1,* *PRICKLE1,* and *MAP3K20* genes, represent potential markers for post-thaw semen quality, and they might be relevant for future improvement in the selection procedure of boars for cryopreservation. The findings of this study provide evidence indicating that polymorphisms in genes expressed in spermatozoa could be considered as factors associated with post-thaw semen quality.

## 1. Introduction

Cryopreservation of semen allows for prolonged storage of genetically important reproductive traits through the use of assisted reproductive techniques (ARTs), such as artificial insemination (AI) [1,2]. Due to poor post-thaw semen quality and reduced fertility, the widespread application of frozen-thawed (FT) boar semen in the AI practices is limited compared to liquid-stored semen [2]. Selection of high proportions of viable FT spermatozoa is the major challenge in the pig AI industry [3]. Moreover, the selection of a high number of functionally viable FT boar spermatozoa is required to minimize economic loss and increase the worldwide application of FT semen in AI technologies [1,2].

Analysis of different sperm attributes has confirmed that cryo-induced damage to spermatozoa differs among individual boars, suggesting varying responses to the freezing–thawing process [4,5]. Evidence has indicated that the freezability of boar semen is influenced by genetic factors [4,6]. Recent developments in high-throughput sequencing techniques, such as transcriptome sequencing (RNA-Seq), have enabled a thorough analysis of gene expression and genetic variations in the pig reproductive tract [7]. Our recent study has confirmed that there are differences in the transcriptome profiles of boar spermatozoa [8]. Furthermore, it has been confirmed that boars with poor freezability ejaculates are characterized by an overexpression of differentially expressed genes (DEGs) that are mainly associated with inflammation and apoptosis, which increase the sperm susceptibility to cryo-induced damage [8]. Previous studies confirmed that genes enriched in the cytokine–cytokine receptor interaction and inflammatory response-related pathways predisposed boar sperm to cryo-induced damage [9,10]. Hence, transcriptome alterations in spermatozoa have been considered to be one of the main factors affecting the cryo-survival of boar semen [8,9,10]. It should be emphasized that variant calling procedure based on RNA-Seq data has been suggested to be an important screening tool to identify polymorphisms in differentially expressed genes [7] and potential genetic markers associated with production traits in the pig industry [11].

Several authors have reported an increasing number of markers related to reproduction traits in pigs [12,13,14]. Advances in molecular genetics have resulted in the identification of several single nucleotide polymorphisms (SNPs) that are associated with the quality parameters of boar spermatozoa (including motility and morphology), and there has been increasing interest to understand the molecular processes that affect the sperm phenotype traits [12,15]. Evidence has shown that polymorphisms in boar sperm could be used as markers for semen quality [12,15,16]. However, to date, there is limited information about the associations of SNPs with post-thaw quality of boar semen. To our knowledge, no study has yet explored the associations between SNP markers and post-thaw quality semen of the Polish large white (PLW) boars. The objectives of this study were to i) identify polymorphisms in candidate genes (CGs) of boar spermatozoa using variant calling analysis of RNA-Seq dataset, ii) identify the functional categories of SNP abundance in the CGs by gene ontology (GO) enrichment analysis, iii) validate the selected SNPs using the Kompetitive Allele Specific Polymorphism (KASP) genotyping assay, and iv) explore the associations of gene polymorphisms with post-thaw (PT) semen quality.

## 2. Results

### 2.1. Assessment of Semen Quality

Repeated measures analysis of variance (ANOVA) did not show significant differences (*p* > 0.05) among the boars, with respect to fresh, pre-freeze semen quality. However, ANOVA results demonstrated that boar was a significant (*p* < 0.001) factor affecting post-thaw motility, mitochondrial membrane potential (MMP), plasma membrane integrity (PMI), normal apical ridge (NAR) acrosome integrity, DNA fragmentation, and lipid peroxidation (LPO). The quality of fresh, pre-freeze, and post-thaw semen for 40 boars is shown in Table 1. Boars showing more than 30% (>30%) total motility were considered as having good semen freezability (GSF), whereas boars with motility less than 30% (<30%) were considered as having poor semen freezability (PSF). Based on post-thaw sperm analysis, the semen of 21 boars was classified as having GSF, whereas the semen of 19 boars was classified as having PSF (Table 1). Besides post-thaw motility, membrane integrity of FT spermatozoa was significantly higher (*p* < 0.05) in boars of the GSF group compared with those of the PSF group (Table 1).

### 2.2. RNA-Seq Mapping and Variant Calling

Clean reads that were mapped to the reference genome ranged from 38,651,853 to 58,239,476, corresponding to about 72.8% uniquely mapped reads (range, 69.8% to 76.7%). The results of the mapping procedure were merged into a single binary alignment map (BAM) file for each boar. The fastq sequence dataset of each library is accessible in the NCBI-SRA database (Bioproject: PRJNA415904; accession number: SRP121647).

Using rMATS-DVR software, in conjunction with the genome analysis tool kit (GATK), a total of 1,389,568 variants were identified in the sperm RNA-Seq datasets of the six boars. Analysis of the filtered/trimmed data base single nucleotide polymorphisms (dbSNPs) resulted in an average of 599,428 raw SNPs, corresponding to approximately 88,340 SNPs (≥10% reads ≥90%) and 82,356 SNPs (≥10% reads = 100%), as shown in Table 2. Following stringent filtering analysis of the dbSNPs, 1371 SNP variants were annotated (Supplementary Table S1), of which about 67% putative polymorphisms (919/1371) were located at the 3’-untranslated regions (3’-UTRs) (Table 3). Furthermore, variant calling detected approximately 5.4% missense polymorphism (74/1371) in genes expressed in boar spermatozoa (Table 3).

### 2.3. SNP Abundance in Functional Categories of Candidate Genes (CGs)

A total of approximately 180 CG genes were enriched in at least 27 pathways, and were mainly distributed in the inflammatory-related pathways followed by the glutamatergic synapse and signaling pathways (Supplementary Table S2). Furthermore, Kyoto Encyclopedia of Genes and Genomes (KEGG) pathway showed a high number of the CG genes were enriched in the PI3K-Akt signaling and metabolic pathways (Supplementary Table S2).

**Figure 1:**
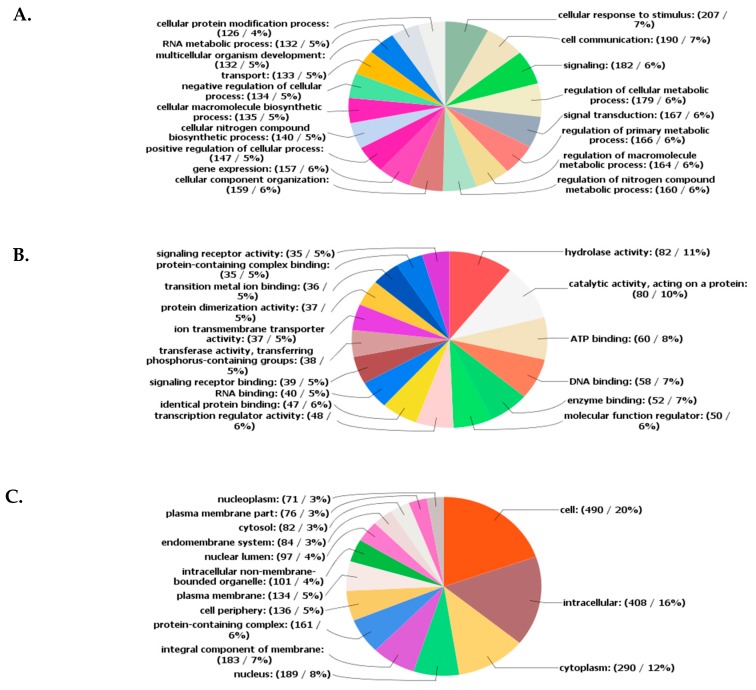
Gene ontology (GO) of enriched terms associated with candidate genes (CGs) in boar spermatozoa. **A**. Biological process categories; **B**. molecular function categories; **C**. cellular component categories. The values in the parenthesis indicate the number of inputs and the percentage of CGs enriched to the category.

Enrichment analysis showed the gene ontology (GO) terms of the CGs (Figure 1, Supplementary Table S3). Generally, the CGs were associated with various functional categories, such as biological process categories (Figure 1A), molecular function categories (Figure 1B), and cellular component categories (Figure 1C). The more prominent enriched terms of the GO biological process categories included “cellular response to stimulus” (*APPL1, TXNIP,* and *OXSR1*) and “cell communication” (*MYO3B*, *EXOC4,* and *KIF1B*), as shown in (Figure 1A). The GO molecular function categories were mainly associated with “hydrolase activity” (*PARG*, *GBA3,* and *GM2A*) and “catalytic activity, acting on a protein” (*HARS2*, *PLBD1,* and *SARS*) (Figure 1B). In addition, the GO cellular component categories included enriched terms associated with “cell” (*CFAP52*, *ANKRD50,* and *SKAP2*) and “intracellular” (*EVA1A*, *TRIM9,* and *PRICKLE1*) (Figure 1C).

The selected CGs for KASP genotyping markers are highly associated with various reproductive processes and sperm functions (Supplementary Table S4). Analysis of the reproduction quantitative traits (QTLs) of the selected CGs showed that age at puberty, corpus luteum number, litter size, and number of stillborns were commonly detected among the pig reproductive traits, according to the Animal Genome pigQTLdb (https://www.animalgenome.org/cgi-bin/QTLdb/SS/index).

### 2.4. KASP Genotyping and Validation

Following KASP genotyping, the allele call rate was 92%, and the genotyping of three SNP markers—*AHI1*, *GLMN,* and *IFNAR2*—was unsuccessful, probably due to poor design of the primers. It was found that *MY3OB* was monomorphic, whereas three other SNP markers—*CDK17*, *CLNK,* and *CRISP2*—showed an excess of homozygotes or heterozygotes in either freezability group. These SNPs were removed from further analysis. Among the SNPs analyzed in this study, the χ2-test and probability estimates showed that most of the SNP loci were within in the Hardy–Weinberg equilibrium (HWE) expectations (Table 4). Noticeable significant departures (*p* < 0.05) from HWE were common in either the GSF group or PSF group, or in both freezability groups, such as at *A2M* g.62485457G > A (rs339026428) and *COMMD2* g.189987893G > A (rs318435440) loci (Table 4).

There were variations in the frequency distributions of the alleles of the CGs (Table 4). Irrespective of the freezability group, higher frequency of allele G was observed in *CYP7B1* (0.90) gene locus (Table 4A), whereas allele A was more frequent in *CFAP52* (0.72) gene locus (Table 4B). Higher frequency of allele C was observed in *EML6* (0.72) gene locus (Table 4C), whereas higher frequency distributions of allele T were observed in *ROBO1* (0.87) gene locus (Table 4F).

**Figure 2:**
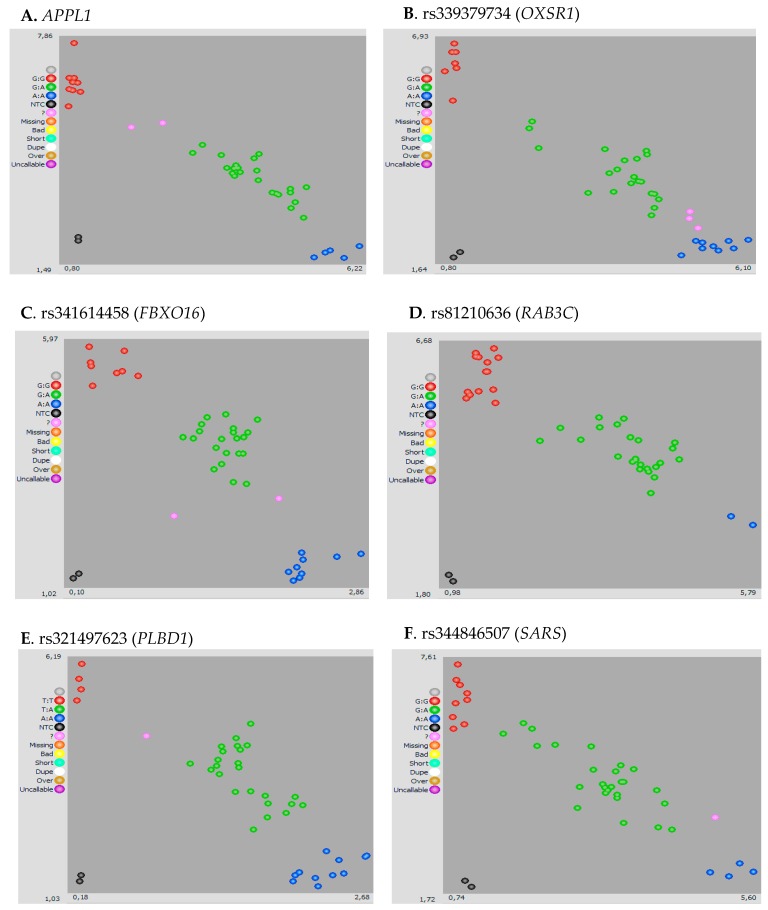
Genotyping cluster plots of **A**. *APPL1*, **B**. *OXSR1*, **C**. *FBXO16,* **D**. *RAB3C*, **E**. *PLBD1*, and **F**. *SARS* genes using the Kompetitive Allele Specific PCR (KASP) assay. Samples shown in pink were considered questionable genotypes as they were not assigned to the cluster. NTC, non-template control. Visualization of the genotype data was performed with the SNPViewer software (http://results.lgcgenomics.com/software/snpviewer).

Genotyping cluster plots of a few genes with polymorphism used for KASP genotyping assay are shown in Figure 2A–F (*APPL1*, *OXSR1*, *FBXO16*, *RAB3C*, *PLBD1*, and *SARS*). Samples shown in pink dots were not assigned to the cluster.

### 2.5. Association Analysis of SNPs with Post-Thaw Semen Quality

ANOVA analysis showed that polymorphisms in *APPL1* (*p* < 0.017), *CYP7B1* (*p* < 0.031), *RAB3C* (*p* < 0.043), *OXSR1* (*p* < 0.047), *FBXO16* (*p* < 0.045), and *PLBD1* (*p* < 0.046) genes had significant effects on post-thaw sperm motility. Polymorphisms in *APPL1* and *PLBD1* genes markedly affected post-thaw PMI (*p* < 0.048 and *p* < 0.025, respectively) and NAR acrosome integrity (*p* < 0.016 and *p* < 0.018, respectively). Also, polymorphism in *PLBD1* gene affected post-thaw MMP and LPO (*p* < 0.018 and *p* < 0.043, respectively). It was observed that polymorphisms in *SARS* gene had marked effects on post-thaw MMP (*p* < 0.046) and NAR acrosome integrity (*p* < 0.001), while *EML5* polymorphism affected post-thaw DNA integrity (*p* < 0.015), LPO (*p* < 0.035), and NAR acrosome integrity (*p* < 0.048). Furthermore, polymorphisms in *RAB3C* and *PRICKLE1* genes had marked effects on post-thaw NAR acrosome integrity (*p* < 0.041 and *p* < 0.040, respectively) and LPO (*p* < 0.037 and *p* < 0.013, respectively). In addition, polymorphisms in *FBXO16* gene affected post-thaw MMP (*p* < 0.048).

**Figure 3:**
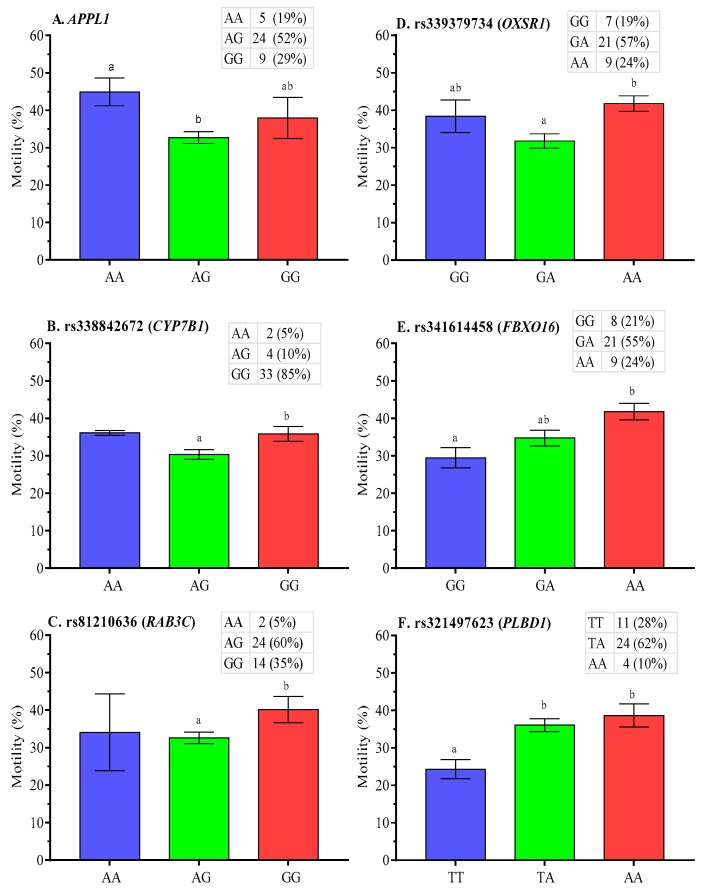
Effect of polymorphisms in **A**. *APPL1*, **B**. *CYP7B1*, **C**. *RAB3C*, **D**. *OXSR1*, **E**. *FBXO16*, and **F**. *PLBD1* genes on post-thaw motility of boar spermatozoa. Data are presented as the mean ± SEM; ^a,b^ Differences between genotypes were significantly differed at *p* < 0.05.

Association analysis of post-thaw semen quality showed that motility was lower (*p* < 0.05) for AG genotype of *APPL1* gene (a newly predicted SNP) (Figure 3A). Likewise, post-thaw motility was lower (*p* < 0.05) for AG genotype (AG vs. GG) at rs338842672 (*CYP7B1* gene) and rs81210636 (*RAB3C* gene) (Figure 3B,C, respectively). Furthermore, post-thaw motility was lower (*p* < 0.05) for the heterozygous genotype at rs339379734 (*OXSR1* gene) compared with AA genotype (Figure 3D), and for GG genotype at rs341614458 (*FBXO16* gene), as shown in Figure 3E. As regards *PLBD1* gene polymorphism, post-thaw motility was the lowest for TT genotype at rs321497623 (Figure 3F).

**Figure 4:**
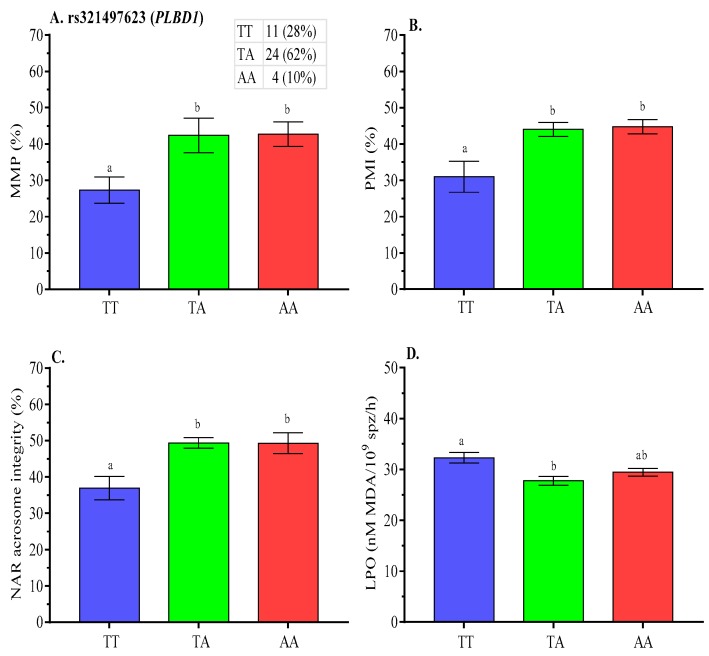
Effect of *PLBD1* gene polymorphism on post-thaw **A**. MMP, **B**. PMI, **C**. NAR acrosome integrity, and **D**. LPO of boar spermatozoa. Data are presented as the mean ± SEM. ^a,b^ Differences between genotypes were significantly differed at *p* < 0.05.

Significantly lower (*p* < 0.05) post-thaw MMP (Figure 4A), PMI (Figure 4B), and acrosome integrity (Figure 4C) were concurrent with higher post-thaw LPO for TT genotype at rs321497623 (Figure 4D).

**Figure 5:**
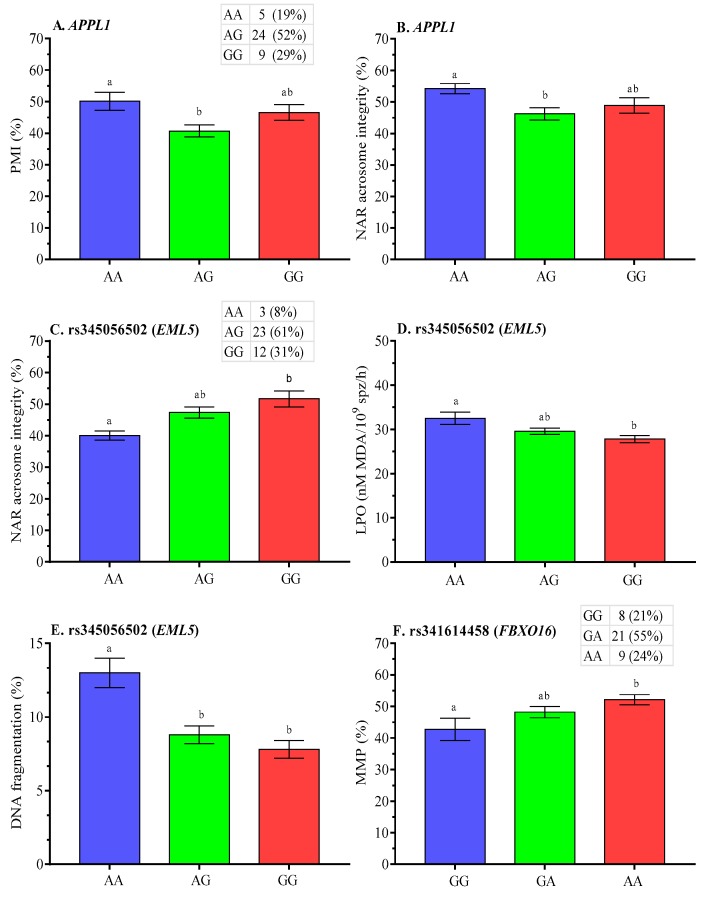
Effect of polymorphisms in **A–B**. *APPL1*, **C**–**E**. *EML5,* and **F**. *FBXO16* genes on post-thaw membrane integrity of boar spermatozoa. Data are presented as the mean ± SEM. ^a,b^ Differences between genotypes were significantly different at *p* < 0.05.

Boars with AG genotype of *APPL1* gene had significantly lower (*p* < 0.05) post-thaw PMI and NAR acrosome integrity (Figure 5A,B, respectively), while lower post-thaw NAR acrosome integrity and higher LPO were observed for AA genotype at rs345056502 (*EML5* gene) (Figure 5C,D, respectively). Furthermore, post-thaw DNA damage was highest (*p* < 0.05) for AA genotype at rs345056502 (Figure 5E), while lower post-thaw MMP was observed for GG genotype at rs341614458 (Figure 5F).

**Figure 6:**
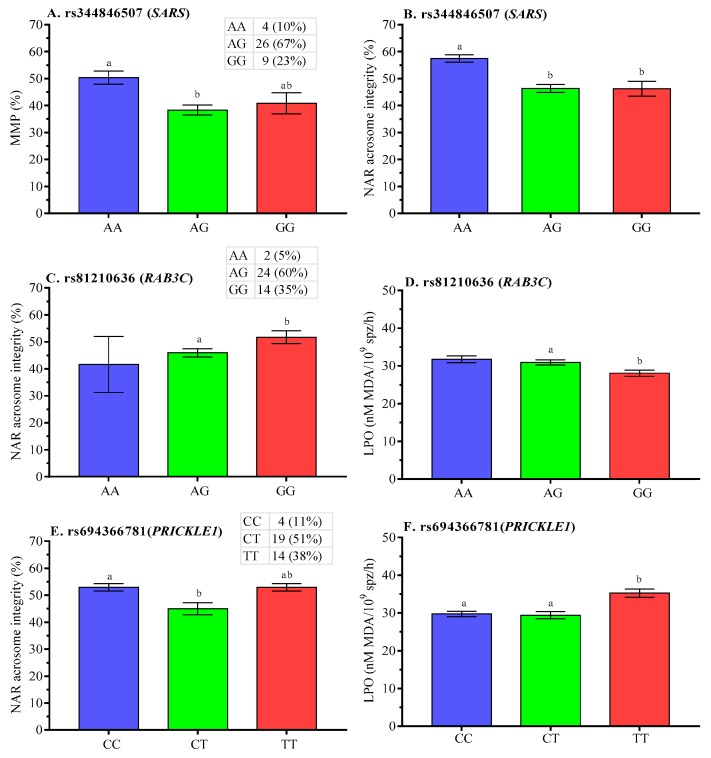
Effect of polymorphisms in **A–B**. *SARS,* **C–D**. *RAB3C,* and **E–F**. *PRICKLE1* genes on post-thaw membrane integrity of boar spermatozoa. Data are presented as the mean ± SEM. ^a,b^ Differences between genotypes were significantly different at *p* < 0.05.

Post-thaw analysis showed that boars with AG genotype had lower (*p* < 0.05) MMP and acrosome integrity at rs344846507 (*SARS* gene) (Figure 6A,B, respectively). It was found that lower (*p* < 0.05) post-thaw NAR acrosome integrity was concomitant with higher LPO for genotype AG (AG vs. GG) at rs81210636 (Figure 6C,D, respectively). Higher post-thaw acrosome damage and LPO were associated with CT or TT genotype at rs694366781 (*PRICKLE1* gene) (Figure 6E,F, respectively).

ANOVA analysis revealed that freezability (F) and SNP genotype (G) and their interactions (F × G) significantly affected (*p* < 0.05) post-thaw semen quality (Table 5). Significant F × G interactions with post-thaw motility and PMI were observed for rs337913978 (*SCLT1* gene, Table 5A). Also, freezability (*p* < 0.001) and F × G interaction (*p* < 0.010) with post-thaw NAR acrosome integrity were observed for rs340643892 (*MAP3K20* gene, Table 5B). Post-thaw LPO and DNA fragmentation showed significant SNP effects and F × G interaction for rs339836492 (*MS4A2* gene) and rs331568674 (*ROBO1* gene), respectively (Table 5C,D, respectively).

**Figure 7:**
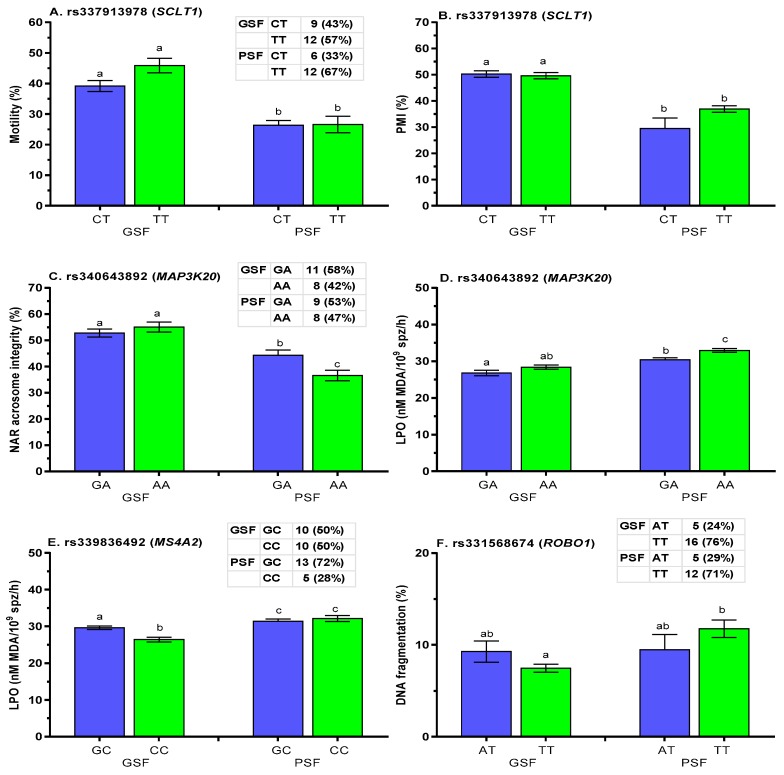
Effect of freezability and gene polymorphisms in **A–B**. *SCLTL1*, **C–D**. *MAP3K20*, **E**. *MS4A2,* and **F**. *ROBO1* genes on post-thaw quality of boar semen. Data are presented as the mean ± SEM. ^a,b^ Differences between genotypes were significantly different at *p* < 0.05; GSF—good semen freezability; PSF—poor semen freezability.

Association analysis of post-thaw sperm quality showed that PSF boars with CT and TT genotypes at rs337913978 (*SCLT1* gene) exhibited lower (*p* < 0.05) motility and PMI (Figure 7A,B, respectively). Significantly (*p* < 0.05) lower proportions of FT spermatozoa with acrosome integrity were concomitant with higher LPO for AA genotype at rs340643892 (*MAP3K20* gene) in boars from the PSF group (Figure 7C,D, respectively). Post-thaw LPO was higher (*p* < 0.05) at rs339836492 (*MS4A2* gene) (Figure 7E), while post-thaw DNA damage was greater (*p* < 0.05) for TT genotype at rs331568674 (*ROBO1* gene) in boars of the PSF group (Figure 7F).

## 3. Discussion

### 3.1. SNP Functional Classes and Validation

In the present study, among the RNA-Seq SNPs (Supplementary Table S1), approximately 82% of the putative polymorphisms in boar spermatozoa had a corresponding dbSNP entry. In a previously published paper, about 88–91% of the detected SNPs in the testis tissue of boars had a dbSNP entry, and a large number of SNPs were at the 3’-UTRs, the untranslated region of noncoding mRNAs [7]. In this study, we found that about 67% of putative polymorphisms were at the 3’-UTR (Table 3), and the impact of most of these polymorphisms on sperm function is not fully understood. Studies have reported that the 3’-UTR plays an important role in the translation efficiency and stability of mRNA [17,18]. It has been reported that UTRs have strong impacts on post-transcriptional regulation of gene expression, suggesting that dysfunction in the UTRs might have a significant effect on gene expression and the associated cellular viability, growth, and development [17,18]. Among the UTR variants at the 3’-UTR, two SNP polymorphisms, rs339379734 (*OXSR1*) and rs337913978 (*SCLT1*), showed significant associations with post-thaw semen quality. Evidence has indicated that SNPs at the 3’-UTR of the targeted mRNA have been the principal elements of microRNA adhesion, and they are associated with semen quality [19]. We suggest that the 3’-UTR SNPs, detected in this study, might play a significant role at the transcription level. Furthermore, most of the coding sequences detected in spermatozoa were synonymous variants, which do not affect protein sequence, but could be in linkage disequilibrium to other causative mutations [7,12] and could have functional effects on mRNA stability and, ultimately, the phenotype traits [20]. In the present study, eight SNP markers that showed significant associations with post-thaw semen quality were synonymous variants (in *CYP7B1, FBXO16, RAB3C, SARS, MAP3K20, PLBD1, EML5,* and *PROCKLE1* genes), which could be linked to causative mutations with functional effects on the reproductive traits in the pig. Furthermore, two missense polymorphisms in *APPL1* and *MS4A2* genes showed significant associations with post-thaw semen quality. Missense variants affect amino acid translation, protein structure and function, and several missense polymorphisms have been shown to affect enzyme activity [21]. It is reasonable to deduce that the missense polymorphisms in *APPL1* (a novel predicted SNP) and *MS4A2* (rs339836492) genes could have a marked effect on their protein expression levels, which could consequently compromise the sperm response to the freezing–thawing conditions.

Quality assessment of a panel of SNP markers was performed by KASP genotyping assay. It is noteworthy that KASP is a novel competitive allele specific PCR, which is mainly based on the amplification of DNA with a thermal cycler using allele specific primers [22]. Due to its low cost and high efficiency, the KASP genotyping assay has been widely used in SNP genotyping studies related to molecular marker-assisted selection breeding [23]. In this study, the genotype frequencies were consistent with the HWE assumptions for most of the analyzed loci. Screening of SNPs for HWE departure is frequently used when performing association studies [24]. There is little consensus on the appropriate *p*-value threshold for the identification of SNPs that violate the HWE assumption in association studies [25]. In the current study, it is unclear why HWE departure was more common in gene loci of individuals from the PSF group. It seems likely that the marked departure from the HWE could be related to long-term artificial selection and breeding of pigs. Several authors suggested that long-term selective breeding for production traits, not associated with reproductive traits, could be attributed to the marked differences in the genotype frequencies [14,26]. Moreover, it has been confirmed that differences in the allele frequencies in AI boars highly selected for a number of traits that are responsible for HWE departure [26]. It is likely that HWE departure is not necessarily caused by the selection of the analyzed traits, but rather other traits under the control of the selected genes or closely related genes [26]. We suggest that further studies are needed to find out whether such a phenomenon is a common feature of polymorphisms in genes expressed in poor freezability ejaculates.

### 3.2. SNP Associations with Post-Thaw Semen Quality

Evidence has shown that the evaluation of various sperm traits rather than a single trait analysis provides a better fertility prediction of FT semen [27,28,29]. Several sperm attributes have been used to assess the post-thaw quality of boar semen, and among these sperm motility is widely used in semen quality assessment because it correlates with fertility outcomes [30,31]. Sperm motility is assessed subjectively or with the computer-assisted semen analysis (CASA) system; however, subjective motility assessment has been shown to provide reliable estimates in association studies [26,32,33]. In the current study, several sperm attributes have been used to monitor the associations of gene polymorphisms with post-thaw quality of boar semen.

The distribution of genotype frequencies differs among the genotype groups, with respect to polymorphisms in the genes related to GO term, “response to stress”, such as *APPL1*, *OXSR1* (rs339379734), *MAP3K20* (rs340643892), and *MS4A2* (rs339836492). Since stress plays an important role in semen cryopreservation [34], the function and localization of these genes are critical in understanding their impact on post-thaw semen quality. In general, the heterogeneous genotype of the *APPL1* gene polymorphism has been associated with reduced post-thaw semen quality. It is noteworthy that the *APPL1* polymorphism is a newly predicted SNP variant detected in boar spermatozoa, and it is very difficult to make comparisons with other studies. The *APPL1* gene is related to several GO terms, such as “signal transduction”, “cell motility”, and “transmembrane transport”, suggesting its significant relevance in sperm function. Accumulating evidence has shown that *APPL1* regulates the function of the adiponectin receptor (AdipoR1), which is implicated in various biological processes in the reproductive system [35,36]. Adiponectin is abundantly found in the tail region of bull spermatozoa, whereas AdipoR1 has been detected mainly in the equatorial and acrosome regions of sperm [35]. Furthermore, a direct role of the AdipoR1 receptor has been suggested in sperm capacitation [36], and adiponectin and its receptors have been associated with motility parameters of ram spermatozoa [37]. These findings reaffirm the important modulating function of the *APPL1* gene on the action of the AdipoR1 receptor in regulating sperm motility. Consistent with this notion, it seems that polymorphisms in the *APPL1* gene might be a contributing factor to compromised function of FT spermatozoa.

The *OXSR1* gene regulates downstream kinases in response to environmental stress, and it plays a role in the Na^+^-K^+^-2Cl^−^ cotransporter (NKCC1) in the testis [38]. Moreover, *OXSR1* is one of the upstream phosphorylators of the NKCC1, which is activated through a phosphorylation-dependent mechanism and is implicated in sperm–oocyte fertilization events [38]. It seems, therefore, that an *OXSR1* gene polymorphism could reduce the abundance of phosphorylated-NKCC1, resulting in reduced sperm motility and compromised IVF potential [38]. Association analysis shows that heterogeneous genotype of *OXSR1* gene significantly increases the sperm susceptibility to reduced post-thaw motility. Hence, it seems likely that the *OXSR1* gene polymorphism compromises the sperm’s ability to confer protection against oxidative stress, particularly during the freezing–thawing process. Cryo-induced stress increases the sperm susceptibility to reactive oxygen species (ROS)-related damage, such as mitochondrial dysfunction, peroxidation of sperm membrane lipids, and DNA damage, resulting in compromised fertility of FT spermatozoa [5,29,39]. Furthermore, it seems that the AA genotype at rs340643892 (*MAP3K20* gene) might predispose spermatozoa to increased LPO and acrosome damage after freezing–thawing. It should be noted that MAPK is a serine/threonine kinase, which is implicated in the activation of gene transcription and expression [40]. Moreover, the MAPK cascade elements have been detected in the flagellum of human spermatozoa and are involved in the regulation of capacitation and the acrosome reaction processes [40]. Analysis shows that GC and CC genotypes at rs339836492 (*MS4A2* gene) were associated with increased post-thaw LPO in poor freezability ejaculates, suggesting that spermatozoa from these ejaculates were more susceptible to increased damage incurred by ROS action during the freezing–thawing process. Although the functions of many of the *MS4A* proteins are currently not well defined, it was reported that the *MS4A* family member could form oligomers in sperm membranes, which might be involved in the interaction with the zona pellucida or cumulus during fertilization [41]. It is noteworthy that the *MS4A2* transcript has been detected in the sperm head [42], suggesting its relevance in sperm–oocyte interaction mechanisms [41]. Our results show that polymorphisms in *MAP3K20* and *MS4A2* genes are associated with reduced post-thaw semen quality, which was manifested mainly in increased susceptibility of FT spermatozoa to LPO, as observed in poor freezability ejaculates. Moreover, an increase in LPO of FT spermatozoa results in a substantial loss of membrane integrity and motility, and ultimately leads to reduced fertility [43]. Additional studies are required to determine the biological relevance of these gene polymorphisms in sperm functions.

Interestingly, it has been confirmed that heterozygous AG (AG vs. GG) of *CYP7B1* gene (rs338842672) is a contributing factor to reduced post-thaw motility. The *CYP7B* gene is enriched in the primary bile acid biosynthesis pathway (ssc00120) and 14 GO terms including “reproduction”, “oxidoreductase activity”, “cell motility”, and “signal transduction”, suggesting the important role of the gene in sperm functions. However, we would like to emphasize that the consequences of *CYP7B1* polymorphism on post-thaw quality of boar semen are not yet known. Similar to the *CYP7B1* gene, only two genotypes (AG vs. GG) of the *RAB3C* gene were considered in the association studies with post-thaw semen quality. In support of this notion, analysis showed that heterozygous AG at rs81210636 (*RAB3C* gene) is also a contributing factor to reduced post-thaw semen quality compared with the GG genotype. The impact of *RAB3C* gene on post-thaw semen quality is unclear. Not much information is available about the role of the *RAB3C* gene in sperm function, but earlier research reported that the *RAB3* protein could interact with a series of proteins and could be associated with the cAMP/PKA (cAMP/protein kinase A) messenger system, in conjunction with phospholipase A_2_ (PLA_2_), to modulate exocytosis of the sperm acrosome [44]. Moreover, the *RAB3* gene is related to 7 GO enriched terms, such as “GTPase activity”, “signal transduction”, and “vesicle-mediated transport”, suggesting the important role of the gene in sperm biological functions. It seems that the higher genotype frequency of heterozygous AG (60%) might predispose FT spermatozoa to compromise fertilization-related events. However, it is unclear how the *RAB3C* protein interacts with boar sperm membrane, and its role in cryo-survival has not yet been elucidated.

In our study, polymorphisms in *FBXO16* (rs341614458) and *PLBD1* (rs321497623) genes were associated with motility and membrane integrity of FT spermatozoa. It should be emphasized that there is not enough information in the literature about the specific role of these genes in the physiological functions of the sperm. It seems that the GG genotype of the *FBXO16* gene is a contributing factor for reduced post-thaw semen quality. The effect of the *FBXO16* polymorphism on sperm function is not fully known; however, members of the F-box family bind to phosphorylated proteins to promote their ubiquitination and degradation, which are required to protect and maintain sperm quality [45,46]. Evidence has shown that ubiquitin-mediated proteolysis of *FBXO* proteins is indispensable for the stability of sperm organelles [45]. Therefore, dysfunction in the ubiquitin-related pathway might have relevance in the physiological functions of the sperm. Furthermore, the effects of *PLBD1* gene polymorphism on the biological function of the sperm is still unclear, even though it has been reported that *PLBD1* is implicated in the fertilization processes [47]. Moreover, the *PLBDI* gene is related to 12 GO enriched terms, such as “lipid metabolic process”, “signal transduction”, and “kinase activity”, suggesting the important role of the gene in various sperm biological functions. It can be suggested that an understanding of the functional significance of *FBXO16* and *PLBD*1 genes is necessary to elucidate their biological effects on sperm functions following cryopreservation.

Analysis showed that polymorphisms in the genes associated with cytoskeleton proteins, namely *EML5* (rs345056502) and *PRICKLE1* (rs694366781), were significantly correlated with reduced motility and acrosome integrity, and increased LPO of FT spermatozoa. It is noteworthy that the cytoskeleton is implicated in various cellular functions including mitosis, membrane translocations, and cellular motility [48]. The impact of polymorphisms of these genes on sperm function is not fully understood. However, our results provide evidence indicating that boars with the homologous AA genotype at rs345056502 (*EML5* gene) were more susceptible to post-thaw acrosome damage and LPO, while the homologous TT genotype of the *PRICKLE1* gene contributed to greater cryo-induced DNA damage. Moreover, microtubule-associated proteins (MAPs) in sperm cells have various functions including modulation of actin cytoskeletal function during spermatogenesis [49]. Collectively, *EML5* and *PRICKLE1* are implicated in microtubule (MT) dynamics in the testis, and mutations in their genotypes could affect the physiological and structural function of the sperm following cryopreservation. Interestingly, no association of either *EML5* or *PRICKLE1* genotypes with post-thaw motility was observed this study. This is surprising due to the critical role of MT or MAPs in the motility apparatus of spermatozoa. A possible explanation could be that the microscope assessment used in this study gives only one measured parameter of sperm motility, while the CASA system provides a variety of different motility parameters [29,50], which might be useful in association studies.

Of the gene associated with t-aminoacylation is *SARS*, polymorphism (rs344846507) in this gene was associated with mitochondrial function and acrosome integrity of FT spermatozoa. It is noteworthy that SARS is a nuclei acid binding gene related to tRNA-aminoacylation biosynthesis (ssc00970) and mitochondrial disorders. Earlier observation showed that increased abundance of *SARS* proteins in spermatozoa resulted in poor blastocyst development [51]. The finding of this study raises the possibility that the *SARS* polymorphism could have an important effect on male fertility, and this necessitates further studies. The *SCLT1* gene is also of importance, in which the frequency distributions of either CT or TT genotype at rs337913978 are associated with reduced post-thaw semen quality. Enrichment analysis showed that the *SCLT1* gene is associated with 9 GO terms, such as “microtubule organizing center” and “cilium”, suggesting the critical role of the gene in sperm function. It has been reported that the *SCLT1* gene polymorphism causes dysfunction in axoneme assembly [52], which could compromise sperm motility. Presently, no significant associations between the *SCLT1* gene and post-thaw semen quality have been reported. Another polymorphism (rs331568674) of interest was detected in the *ROBO1* gene. Our findings show that the *ROBO1* gene is enriched in the axon guidance pathway (ssc04360) and 12 GO terms involved in biological functions, such as “cell motility”, “signal transduction”, and “protein modification process”. In a recent study, it has been reported that the axon guidance pathway has a potential biological function in boar fertility [53], and its pathway has a significant role in DNA methylation alterations in sperm cell development [54]. In the current study, it seems that the TT genotype of the *ROBO1* gene polymorphism might increase the sperm susceptibility to cryo-induced DNA fragmentation, as shown in the poor freezability ejaculates. It should be stressed that the homologous genotype was not detected in *SCLT1* (CT-TT), *MAP3K20* (GA-AA), *MS4A2* (GC-CC), and *ROBO1* (AT-TT) genes. Previous studies reported that the absence of a homologous genotype might indicate its removal through artificial selection and breeding [32,33]. Artificial selection and breeding is constantly practiced in the pig AI industry, and it is possible that such phenomenon might explain the absence of the homologous genotype in the above-mentioned gene polymorphisms. Furthermore, we are unable to explain the presence of low frequency of alleles in the homologous genotype of genes, such as *CYP7B1* and *RAB3C*. Besides intensive selective breeding, it has been suggested that some genotypes might be eliminated by adaption of individuals to environmental stress [33]. However, it is unclear at this point whether the gene polymorphisms that were associated with post-thaw semen quality present a direct functional effect or if they are in linkage disequilibrium with other functional SNPs. Previous studies have reported that the effect on genotype or SNP variant might be influenced by the other SNPs, suggesting the interactions of multiple SNPs on the semen quality traits [32,33]. Accordingly, polymorphisms in boar spermatozoa could be used as markers associated with causative mutations within the gene [12,26].

In this study, the variant calling procedure detects polymorphisms associated with fertilization-related traits based on their relevance in sperm motility, metabolism, reproduction, and embryo development. We described here the associations of sperm-related genetic polymorphisms with post-thaw quality of boar semen. Candidate genes with significant SNP associations, including *APPL1, PLBD1, FBXO16, EML5, RAB3C*, *OXSR1, PRICKLE1,* and *MAP3K20* genes, are promising markers for post-thaw semen quality, and that they might be relevant for future improvement in the selection procedure of boars for cryopreservation. We suggest that polymorphisms in genes expressed in spermatozoa could be considered as factors associated with post-thaw semen quality. However, further well-designed studies, with a larger animal population, are required to investigate the effect of gene polymorphisms in boar spermatozoa on semen freezability.

## 4. Materials and Methods

### 4.1. Animals and Semen Collections

A total of forty PLW boars were used in this study. The six boars used for RNA-Seq were stationed at the Cryopreservation laboratory, Faculty of Animal Bioengineering, University of Warmia and Mazury in Olsztyn [8]. For the association studies, most of the ejaculates were collected from boars, which were stationed at the Cryopreservation laboratory, and a total of 51 ejaculates were collected from boars stationed at three AI centers [5]. A total of approximately 296 ejaculates, at least three ejaculates per boar, were collected from the 40 boars (during the autumn–winter period), using the gloved-hand technique. The animals were fed with a commercial porcine ration and were kept in individual pens throughout the experimental period. Water was available ad libitum. Only sperm samples with more than 70% motility and 85% normal morphology were used for cryopreservation. Animal experiments were carried out in accordance with the guidelines set out by the Local Ethics Committee, Olsztyn (Poland). Experiments on boars (semen collection procedures) do not require the approval of the Local Ethics Committee (Olsztyn) from 15/01/2015.

### 4.2. Cryopreservation Procedure

Semen was frozen according to a cryopreservation protocol using lyophilized lipoprotein fractions of ostrich egg yolk (LPFo), as described in previous studies [39,50,55]. The LPFo-extended semen was cooled to 5 °C for 2h and diluted (2:1) with another freezing extender (89.5 mL lactose-LPFo extender, 9 mL glycerol, and 1.5 mL Orvus Es Paste). All samples (500 × 10^6^ spermatozoa/mL) were frozen in a programmable computer freezer (Ice Cube 1810, SY-LAB, Austria), using an appropriate cooling rate [55], prior to storage in liquid nitrogen. Frozen samples were thawed in a water bath for 60 sec at 50 °C for post-thaw sperm analysis (motility and membrane integrity). Following post-thaw, the samples (50 × 10^6^ spermatozoa/mL) were held in a water bath for 10 min at 37 °C prior to semen quality assessment.

### 4.3. Semen Quality Assessment

Quality assessment was performed in fresh, pre-freeze, and post-thaw semen.

#### 4.3.1. Sperm Motility

The percentages of motile spermatozoa were assessed by the same technician throughout the study. Briefly, semen samples (6 μL) were placed on pre-warmed slide and assessed under a light microscope at 200 × magnification (Olympus BX 40, Tokyo, Japan) equipped with an attached heated stage (37 °C). Sperm motility was evaluated randomly in at least five fields per sample [5].

#### 4.3.2. Mitochondrial Membrane Potential (MMP)

Sperm MMP was assessed with the fluorescent lipophilic cation JC-1 and propidium iodide (PI) fluorescent dyes [5,56].

#### 4.3.3. Plasma Membrane Integrity (PMI)

Sperm PMI was assessed with the SYBR-14 and PI fluorescent probes, using the Live/Dead Sperm Viability Kit (Molecular Probes, Eugene, OR, USA) [57].

#### 4.3.4. Normal Apical Ridge (NAR) Acrosome Integrity

A staining protocol was used to assess the sperm normal apical ridge (NAR) acrosome integrity [34,50].

#### 4.3.5. DNA Fragmentation

The procedure used to assess sperm DNA fragmentation has been described in previous studies [39,55]. Briefly, agarose-embedded sperm samples on microscopic slides were stained with ethidium bromide and were assessed at 400× magnification under a fluorescence microscope (Olympus BX 41, Tokyo, Japan).

#### 4.3.6. Lipid Peroxidation (LPO)

Sperm LPO was determined spectrophotometrically by malondialdehyde (MDA) production [5]. The LPO was defined as the production of nM MDA by 1 × 10^8^ spermatozoa following 1 h incubation at 37 °C (nM MDA/10^8^ spz/h).

### 4.4. Genomic DNA Isolation

Genomic DNA was isolated from washed sperm cells (200 × 10^6^ spermatozoa/mL) of 40 boars (21 boars with GSF and 19 boars with PSF) using the protocol of the Sherlock AX Purification kit (A&A Biotechnology, Gdynia, Poland), according to the manufacturer’s instructions. Lysing solution containing proteinase K and dithiothreitol was added to sperm pellets. The mixture was incubated for 60 min at 50 °C and was subjected to a column filtration and purification procedure. Genomic DNA was treated with a precipitation enhancer isopropanol, and the DNA pellets were washed 2× in 70% ethanol and air-dried (10 min) at room temperature. The isolated DNA samples were dissolved in Tris-EDTA buffer and stored at −20 °C, until further analysis.

### 4.5. Variant Calling and Filtering Analysis

We used the RNA-Seq datasets that were submitted to the National Center for Biotechnology Information (NCBI) Sequence Read Archive (SRA) database (Bioproject: PRJNA415904; accession number SRP121647) (2018/05/27). The datasets represent RNA-Seq from three PLW boars each of the GSF and PSF groups [8]. 

For the RNA-Seq, the clean reads were mapped to the *Sus scrofa* reference genome from Ensembl (genome-build *Scrofa* 11.1.91) using the two-pass mapping strategy in the spliced transcripts alignment to a reference (STAR) software [58]. The BAM alignment files were processed, and single nucleotide variants (SNVs) were called by the Picard tool (http://broadinstitute.github.io/picard) and GATK [59]. The data were filtered based on read depth greater than 10 (≥10) and quality score greater than 90 (≥ 90), as described in a previous study [23]. Comparison of frequency variations of SNVs was performed using the rMATS-DVR software [60]. Each potentially heterozygous genomic position was statistically examined using the False Discovery Rate (FDR< 0.05) to identify variations in the alternate allele frequencies between boars of the freezability groups. The parameters used to identify each SNV in the freezability groups were as follows: gene position, position of *Sus scrofa* chromosome (SSC), accession number in dbSNP database (version 11.91), differences in genetic coverage of both reference and alternate allele counts, allele fraction differences, number of counts for the reference and allele fractions, and the FDR value. Validation of SNPs was performed by matching putative polymorphisms to known pig dbSNP entries using standalone BLAT v.36 [61]. The SNPs that were not present in the dbSNP were considered as unknown (novel predicted SNP). As an additional variant filtering procedure, we retained SNPs that were on the coding sequences (CDS), 3′-untranslated regions (3′-UTRs), 5′untranslated regions (5′-UTRs), putative promotor regions (approximately 200 bp from the beginning of the transcript), and non-coding sequences (ncRNAs). After multi-filtering analysis, the identified variants were annotated by the SnpEff software v.4.1 [62] and Variant Effect Predictor (VEP) Ensembl [63] to retrieve the significant SNPs (Supplementary Table S1). We searched the literature to identify the functions of the CGs in the reproductive processes, sperm physiology or QTLs, according to the Animal Genome pigQTLdb. The CGs containing variants were selected according to their polymorphism coverage in the dataset (Supplementary Table S1) or their role in reproductive processes, sperm functions, or reproduction traits based on QTLs. Thus, by considering these criteria, forty CGs with polymorphisms (Supplementary Table S4) were selected for validation using the KASP genotyping assay (LGC Genomics Ltd., Trident Industrial Estate, Hoddesdon, Hertfordshire, UK).

### 4.6. KEGG Pathways and GO Enrichment Analysis

The Kobas functional annotation tool (v3.0) was used to identify in the Kyoto Encyclopedia of Genes and Genomes (KEGG) pathways statistical enrichment (*p* < 0.05) of the CG genes with polymorphisms [64]. Blast2Go Pro software, v.5.2.5 [65] was used to perform functional annotations, according to gene ontology (GO) categories (biological process, molecular function, and cellular components). *Sus scrofa* Ensembl database was downloaded from Ensembl BioMart Martview application to perform blastx, Blast2GO mapping, and the GO enrichment analysis. The GO significance levels (*p* < 0.05) were computed for multiple testing in the Blast2GO software program [65].

### 4.7. SNP KASP Genotyping Assay

The accuracy of the genotype calls was validated using the KASP genotyping assay (LGC Genomics Ltd., Trident Industrial Estate, Hoddesdon, Hertfordshire, UK). Forty SNPs were genotyped using 50 up and downstream flanking regions (Supplementary Table S5). Genomic DNA isolated from spermatozoa of forty PLW boars was shipped to the LGC Genomics Lab (http://www.lgcgroup.com) to perform the SNP genotyping assay based on KBioscience’s Kompetitive allele-specific PCR amplification. Primers designed for KASP™ genotyping procedure were performed by the LGC Genomics laboratory using the KBioscience PrimerPicker software [22]. The data were analyzed with the Kluster-caller software and SNPViewer (LGC Genomics, Ltd.; http://results.lgcgenomics.com/software/snpviewer,) to identify SNP genotypes.

### 4.8. Statistical Analysis

Data were statistically analyzed with the IBM SPSS Statistics 25 software package (IBM SPSS Statistics for Windows, version 25.0, IBM Corp., Armonk, NY, USA) and Statistica software package, version 12.5 (StatSoft Incorporation, Tulsa, OK, USA). The normality of the data distribution was analyzed with ANOVA assumption (Shapiro Wilk test), and the Levene’s test was used to check for homogeneity of variance. The general linear modeling (GLM) procedure was used for ANOVA analysis. The effect of boar on fresh, pre-freeze, and post-thaw semen quality was analyzed using Model (1).

Y_ij_ = µ +B_i_ + e_ij_ (Model 1)

where Y is the measured semen quality traits; µ is the overall mean; B_i_ is the fixed effect of boar; e_ij_ is the random residual effect.

The effect of SNP (G) on post-thaw semen quality was analyzed using Model (2).

Y_ij_ =µ +G_i_ +e_ij_ (Model 2)

where Y is the measured semen quality traits; µ is the overall mean; G_i_ is the fixed effect of the genotype; e_ij_ is the random residual effect.

In the boar population not all possible genotypes were detected for *SCLT1*, *MAP3K20*, *MS4A2,* and *ROBO1*. The association analysis of these gene polymorphisms with post-thaw semen quality was performed using Model (3).

Y_ijk_ =µ + F_i_ + G_j_ + e_ijk_ (Model 3)

where Y is the measured semen quality traits; µ is the overall mean; F_i_ is the fixed effect of freezability; G_j_ is the fixed effect of the genotype; e_ijk_ is the random residual effect.

Pairwise comparisons were analyzed with an independent two-tailed T-test, while multiple comparisons were performed with Tukey’s honest significant difference (HSD) test as appropriate. Descriptive variables are presented as the mean ± SEM. Significant differences were considered at *p* < 0.05.

The GENEPOP v.4.7.2 software package [66] (https://kimura.univ-montp2.fr/~rousset/Genepop.htm) was used to a) calculate the allele and genotype frequencies (Fisher’s exact test; b) determine Hardy–Weinberg equilibrium (HWE) for all loci using both the Fisher’s exact test and chi-square test (χ2-test), and c) examine the population differences between the two freezability groups.

## Figures and Tables

**Table 1:** Fresh, pre-freeze, and post-thaw quality of boar semen. Data are presented as the mean ± SEM of 21 and 19 boars with good and poor semen freezability (GSF and PSF, respectively).

Sperm Parameters	Fresh, Pre-Freeze Semen	Post-Thaw Semen		Total
		GSF	PSF	GSF + PSF
Motility (%)	75.78 ± 0.28	42.96 ± 1.63 ^a^	26.85 ± 1.28 ^b^	35.31 ± 1.65
MMP (%)	87.83 ± 2.64	48.82 ± 1.04 ^a^	31.55 ± 1.55 ^b^	40.62 ± 1.64
PMI (%)	87.43 ± 1.22	49.91 ± 0.84 ^a^	34.55 ± 1.62 ^b^	42.64 ± 1.51
NAR acrosome integrity (%)	91.62 ± 1.07	53.93 ± 0.60 ^a^	40.85 ± 0.86 ^b^	47.72 ± 1.38
DNA fragmentation (%)	2.97 ± 0.13	7.85 ± 0.45 ^a^	10.56 ± 0.83 ^b^	9.16 ± 0.50
LPO (nMol MDA/10^9^spz/h)	21.34 ± 0.68	27.37 ± 0.47 ^a^	31.55 ± 0.48 ^b^	29.36 ± 0.45

Values with different letters (a and b) are significantly differed (*p* < 0.05); MMP—mitochondrial membrane potential; PMI—plasma membrane integrity; NAR—normal apical ridge; LPO—lipid peroxidation; MDA—malondialdehyde.

**Table 2:** Analysis of filtered and trimmed dbSNPs in boar spermatozoa using variant calling of RNA-Seq datasets.

Sample ID	Raw SNV	≥10% Reads ≥90%	% Raw Reads	≥10 Reads = 100%	Raw dbSNPs	≥10% Reads ≥90%	% Raw Reads	≥10 Reads = 100%
G01	941,355	80,714	8.6%	66,625	632,893	68,577	10.8%	57,182
G09	873,327	78,084	8.9%	73,323	583,610	66,131	11.3%	62,796
G17	941,859	144,415	15.3%	135,223	618,586	122,917	19.9%	116,302
P30	866,451	100,806	11.6%	94,399	575,549	84,148	14.6%	79,695
P38	890,984	110,574	12.4%	103,347	591,527	94,160	15.9%	88,990
P39	898,293	110,753	12.3%	103,664	594,402	94,107	15.8%	89,169

dbSNP—data base single nucleotide polymorphism; SNV—single nucleotide variant.

**Table 3:** Putative SNPs detected in boar spermatozoa.

SNP Effect	SNP Count	Percent (%)
3 prime UTR variant	919	67.031
5 prime UTR variant	120	8.752
Synonymous variant	100	7.294
Missense variant	74	5.398
Non coding transcript exon variant	57	4.158
Splice region variant	4	0.292
Intron variant, non-coding transcript variant	3	0.219
Stop gained	2	0.145
Others	4	0.292
Unknown	88	6.419

**Table 4:** Genic differentiation, frequencies of single nucleotide polymorphism (SNP) alleles, and Hardy–Weinberg equilibrium (HWE) with respective *p*-value, chi-square (χ2), and probability. GSF—good semen freezability; PSF—poor semen freezability.

Table 4 **A**											
**SNP ID**	**Locus**	**Freezability** **Groups**	**Allele Counts**		**Total**	**Allele Frequencies**		***p*-value**	**HWE**		
			**A**	**G**		**A**	**G**		***p*-value**	**χ2**	**Probability**
	*APPL1*	GSF	19	23	42	0.452	0.548	1.00000	1.0000	5.8659	0.2094
unknown		PSF	15	19	34	0.441	0.559		0.0532		
		Total	34	42	76	0.447	0.553				
	*CYP7B1*	GSF	5	35	40	0.125	0.875	0.71180	0.0105	9.1044	0.0585
rs338842672		PSF	3	35	38	0.079	0.921		1.0000		
		Total	8	70	78	0.103	0.897				
	*EML5*	GSF	12	30	42	0.286	0.714	0.06221	0.1283	6.1640	0.1872
rs345056502		PSF	17	17	34	0.500	0.500		0.3575		
		Total	29	47	76	0.382	0.618				
	*LPAR1*	GSF	26	14	40	0.650	0.350	0.62845	0.6347	2.0228	0.7316
unknown		PSF	24	10	34	0.706	0.294		0.5731		
		Total	50	24	74	0.676	0.324				
	*RAB3C*	GSF	11	31	42	0.262	0.738	0.10038	1.0000	7.5732	0.1085
rs81210636		PSF	17	21	38	0.447	0.553		0.0227		
		Total	38	52	80	0.350	0.650				
	*SARS*	GSF	19	21	40	0.475	0.525	0.50110	1.0000	8.8484	0.0650
rs344846507		PSF	15	23	38	0.395	0.605		0.0120		
		Total	34	44	78	0.436	0.564				
	*TXNIP*	GSF	12	30	42	0.286	0.714	0.10631	0.1301	14.0970	0.0070
rs340075321		PSF	18	20	38	0.474	0.526		0.0067		
		Total	30	50	80	0.375	0.625				
Table 4 **B**											
**SNP ID**	**Locus**	**Freezability** **Groups**	**Allele Counts**		**Total**	**Allele Frequencies**		***p*-** **value**	**HWE**		
			**G**	**A**		**G**	**A**		***p*-value**	**χ** **2**	**Probability**
	*OXSR1*	GSF	18	24	42	0.429	0.571	0.48138	0.3775	8.4171	0.0774
rs339379734		PSF	17	15	32	0.531	0.469		0.0394		
		Total	35	39	74	0.473	0.527				
	*A2M*	GSF	8	34	42	0.190	0.810	0.36658	0.0001	26.9432	0.0000
rs339026428		PSF	2	36	38	0.053	0.947		0.0271		
		Total	10	70	80	0.125	0.875				
	*ANKRD42*	GSF	16	26	42	0.381	0.619	0.49879	0.6413	18.6650	0.0009
rs81210697		PSF	20	18	38	0.526	0.474		0.0015		
		Total	36	44	80	0.450	0.550				
	*CCDC149*	GSF	17	25	42	0.405	0.595	0.48804	0.3681	7.5901	0.1078
rs332902509		PSF	17	17	34	0.500	0.500		0.0611		
		Total	34	42	76	0.447	0.553				
	*CFAP52*	GSF	28	12	40	0.700	0.300	0.79820	0.6178	3.5245	0.4742
unknown		PSF	25	9	34	0.735	0.265		0.2779		
		Total	53	21	74	0.716	0.284				
	*COMMD2*	GSF	19	21	40	0.475	0.525	0.64518	0.0050	19.7578	0.0006
rs318435440		PSF	14	20	34	0.412	0.588		0.0102		
		Total	33	41	74	0.446	0.554				
	*FBXO16*	GSF	16	24	40	0.400	0.600	0.16668	1.0000	0.9449	0.9180
rs341614458		PSF	21	15	36	0.583	0.417		0.6235		
		Total	37	39	76	0.487	0.513				
	*MAP3K20*	GSF	11	27	38	0.289	0.711	1.00000	0.2531	5.3360	0.2545
rs340643892		PSF	9	25	34	0.265	0.735		0.2741		
		Total	20	52	72	0.278	0.722				
	*WRN*	GSF	9	29	38	0.237	0.763	0.76470	1.0000	0.0000	1.0000
rs319208708		PSF	5	21	26	0.192	0.808		1.0000		
		Total	14	50	64	0.212	0.788				
Table 4 **C**											
**SNP ID**	**Locus**	**Freezability** **Groups**	**Allele Counts**		**Total**	**Allele Frequencies**		***p*-value**	**HWE**		
			**A**	**C**		**A**	**C**		***p*-value**	**χ** **2**	**Probability**
	*CLEC7A*	GSF	15	27	42	0.357	0.643	1.00000	0.6647	3.2122	0.5230
rs325939188		PSF	13	21	34	0.382	0.618		0.3019		
		Total	28	48	76	0.368	0.632				
	*EML6*	GSF	10	30	40	0.250	0.750	0.60588	1.0000	4.4370	0.3501
rs322659685		PSF	11	23	34	0.324	0.676		0.3501		
		Total	21	53	73	0.284	0.716				
			**C**	**A**		**C**	**A**				
	*ABCB11*	GSF	16	24	40	0.400	0.600	0.62897	0.0005	15.2834	0.0041
rs324930519		PSF	11	23	34	0.324	0.676		1.0000		
		Total	27	47	74	0.635	0.365				
	*SMS*	GSF	12	26	38	0.316	0.684	0.33352	0.0015	13.8162	0.0079
rs343122214		PSF	16	20	36	0.444	0.556		0.6577		
		Total	28	46	74	0.378	0.622				
Table 4 **D**											
**SNP ID**	***Locus***	**Freezability Group**	**Allele Counts**		**Allele Frequencies**		**Total**	***p*-value**	**HWE**		
			**C**	**G**	**C**	**G**			***p*-value**	**χ** **2**	**Probability**
	*TMEM177*	GSF	21	17	38	0.553	0.447	0.81666	0.0001	23.0661	0.0001
rs80954196		PSF	18	18	36	0.500	0.500		0.1750		
		Total	39	35	74	0.527	0.473				
			**G**	**C**		**G**	**C**				
	*MS4A2*	GSF	10	30	40	0.250	0.750	0.32355	0.2784	9.0353	0.0602
rs339836492		PSF	13	23	36	0.361	0.639		0.0392		
		Total	23	53	76	0.303	0.697				
Table 4 **E**											
**SNP ID**	**Locus**	**Freezability Groups**	**Allele Counts**		**Total**	**Allele Frequencies**		***p*-value**	**HWE**		
			**C**	**T**		**C**	**T**		***p*-value**	**χ2**	**Probability**
	*PRICKLE1*	GSF	15	27	42	0.357	0.643	1.00000	1.0000	2.2918	0.6823
rs694366781		PSF	12	20	32	0.375	0.625		0.3179		
		Total	27	47	74	0.365	0.635				
	*SCLT1*	GSF	9	33	42	0.214	0.786	0.77416	0.5322	1.2614	0.8679
rs337913978		PSF	6	30	36	0.167	0.833		1.0000		
		Total	15	63	78	0.192	0.808				
			**T**	**C**		**T**	**C**				
	*ACSL4*	GSF	5	37	42	0.119	0.881	0.36037	1.0000	0.0000	1.0000
rs334625232		PSF	8	28	36	0.222	0.778		1.0000		
		Total	13	65	78	0.167	0.833				
	*ATP5F1A*	GSF	22	20	42	0.524	0.476	0.06697	0.0000	>43.1295	<0.0000
rs328079913		PSF	28	10	38	0.737	0.263		0.0036		
		Total	50	30	80	0.625	0.375				
	*HSPA13*	GSF	11	19	30	0.367	0.633	0.57756	0.2960	3.7925	0.4348
rs335938037		PSF	8	22	30	0.267	0.733		0.5072		
		Total	19	41	60	0.317	0.683				
	*PAM*	GSF	11	29	40	0.275	0.725	0.77866	0.2555	2.7292	0.6041
rs81217594		PSF	6	20	26	0.231	0.769		1.0000		
		Total	17	49	66	0.258	0.742				
	*RIOX2*	GSF	9	29	38	0.237	0.763	0.57428	1.0000	0.0000	1.0000
unknown		PSF	8	18	26	0.308	0.692		1.0000		
		Total	17	47	64	0.266	0.734				
	*SKAP2*	GSF	16	26	42	0.381	0.619	0.21618	0.6422	2.1935	0.7002
rs336351767		PSF	8	26	34	0.235	0.765		0.5200		
		Total	24	52	76	0.316	0.684				
Table 4 **F**											
**SNP ID**	**Locus**	**Freezability group**	**Allele counts**		**Total**	**Allele frequencies**		***p*-value**	**HWE**		
			**A**	**T**		**A**	**T**		***p*-value**	**χ2**	**Probability**
	*ROBO1*	GSF	5	37	42	0.119	0.881	0.74597	1.0000	0.0000	1.0000
rs331568674		PSF	5	29	34	0.147	0.853		1.0000		
		Total	10	66	76	0.132	0.868				
			**T**	**A**		**T**	**A**				
	*PLBD1*	GSF	14	28	42	0.333	0.667	0.16654	0.0487	6.0432	0.1959
rs321497623		PSF	18	18	36	0.500	0.500		1.0000		
		Total	32	46	78	0.410	0.590				
Table 4 **G**											
**SNP ID**	**Locus**	**Freezability group**	**Allele counts**		**Total**	**Allele frequencies**		***p*-value**	**HWE**		
			**G**	**T**		**G**	**T**		***p*-value**	**χ2**	**Probability**
	*HARS2*	GSF	8	32	40	0.200	0.800	0.78401	0.5482	2.5152	0.6419
rs336003721		PSF	8	26	34	0.235	0.765		0.5187		
		Total	16	58	74	0.216	0.784				

**Table 5:** ANOVA analysis showing the effects of freezability (F) and gene polymorphism (G) on post-thaw semen quality.

**A**. rs337913978 (*SCLT1*)	**Motility**			**Plasma Membrane Integrity (PMI)**		
	**F**	**G**	**F × G**	**F**	**G**	**F × G**
	*p*-value	*p*-value	*p*-value	*p*-value	*p*-value	*p*-value
	<0.001	>0.305	<0.043	<0.001	>0.063	<0.028
**B.** rs340643892 (*MAP3K20*)	**NAR Acrosome Integrity**			**Lipid Peroxidation (LPO)**		
	**F**	**G**	**F × G**	**F**	**G**	**F × G**
	*p*-value	*p*-value	*p*-value	*p*-value	*p*-value	*p*-value
	<0.001	>0.146	<0.010	<0.001	<0.022	>0.981
**C.** rs339836492 (*MS4A2*)	**Lipid Peroxidation (LPO)**					
	**F**	**G**	**F × G**			
	*p*-value	*p*-value	*p*-value			
	<0.001	<0.039	>0.302			
**D.** rs331568674 (*ROBO1*)	**DNA Fragmentation**					
	**F**	**G**	**F × G**			
	*p*-value	*p*-value	*p*-value			
	<0.030	>0.800	<0.047			

Significantly differed at *p* < 0.05; NAR—normal apical ridge.

## Data Availability

Raw sequencing data in the fastq format were submitted to the National Center for Biotechnology Information (NCBI) SRA database (https://www.ncbi.nlm.nih.gov/Traces/sra), with accession number SRP121647.

## Supplementary Materials

Supplementary materials can be found at https://www.mdpi.com/1422-0067/21/5/1902/s1. Supplementary Table S1. Identification of single-nucleotide variants (SNVs) in spermatozoa boars with good and poor semen freezability (GSF and PSF, respectively). Supplementary Table S2. KEGG pathway of enriched candidate genes (CGs) of boar spermatozoa. Supplementary Table S3. Gene Ontology (GO) of enriched terms associated with selected candidate genes (CGs) of boar spermatozoa. Table S4. Reproductive processes, sperm functions and quantitative trait loci (QTLs) for pig reproductive traits of selected candidate genes (CGs) with single nucleotide polymorphisms (SNPs). Supplementary Table S5. Single nucleotide polymorphism (SNP) markers and primer sequences used for KASP genotyping assay. References [67,68,69,70,71,72,73,74,75,76,77,78,79,80,81,82,83,84,85,86,87,88,89,90,91,92] are cited in the supplementary materials.
