# Supplementary material for: Gene Polymorphisms in Boar Spermatozoa and Their Associations with Post-Thaw Semen Quality
Source: Int J Mol Sci. 2020 Mar 10;21(5):1902. doi: 10.3390/ijms21051902 (PMC7084667; doi:10.3390/ijms21051902)
Supplement: Supplementary file 1 [file ijms-21-01902-s001.zip › Supplementary Table S4.docx]

**Supplementary Table S4**. Reproductive processes, sperm functions and quantitative trait loci (QTLs) for pig reproductive traits of selected candidate genes (CGs) with single nucleotide polymorphisms (SNPs).

| **SNP** | ***Name** | **Reproductive processes/sperm functions** | **References** | **QTLs** |
| --- | --- | --- | --- | --- |
| unknown | *APPL1* | Apoptosis, motility and capacitation | [35] | age at puberty |
| rs339379734 | *OXSR1* | Phosphorylation, spermatogenesis and fertilization | [38] | age at puberty, number of stillborn |
| rs340643892 | *MAP3K20* | Sperm-oocyte interaction | [40] | number of stillborn |
| rs339836492 | *MS4A2* | Sperm-oocyte interaction | [41] | - |
| rs341011509 | *GLMN* | Unclear defined function in spermatozoa. | Acc:100153954; [42] | - |
| rs325770408 | *CLNK* | Signal transduction | Acc:HGNC:17438; [42] | - |
| rs81210636 | *RAB3C* | Sperm-oocyte interaction | [44] | - |
| rs341614458 | *FBXO16* | Maintenance of sperm function | [46] | litter size |
| rs321497623 | *PLBD1* | Sperm-oocyte interaction | [47] | reproductive tract weight |
| rs694366781 | *PRICKLE1* | Spermatogenesis. | [49] | number of stillborn |
| rs344846507 | *SARS* | Embryo development | [51] | - |
| rs337913978 | *SCLT1* | Spermatogenesis | [52] | age at puberty, litter size |
| rs332902509 | *CCDC149* | Sperm morphology | [53] | litter size |
| rs338842672 | *CYP7B1* | Steroidogenesis and metabolism | [67] | corpus luteum number |
| rs345056502 | *EML5* | Spermatogenesis | [68] | - |
| unknown | *LPAR1* | Spermatogenesis and signal transduction | [69] | - |
| rs340075321 | *TXNIP* | Motility, capacitation and DNA stability | [70] | corpus luteum number |
| rs80886473 | *CRISP2* | Motility and Sperm-oocyte interaction | [71] | corpus luteum number |
| unknown | *IFNAR2* | Immune-related response. | [72] | - |
| rs196959943 | *AHI1* | Spermatogenesis. | [73] | - |
| rs339026428 | *A2M* | Epididymal sperm maturation. | [74] | number of stillborn |
| rs81210697 | *ANKRD42* | Motility and acrosome reaction. | [75] | embryo survival |
| unknown | *CFAP52* | Stabilization of axoneme structure in spermatozoa. | [76] | - |
| rs318435440 | *COMMD2* | Post-translation protein modifications | Acc:100522759 | - |
| rs319208708 | *WRN* | Spermatogenesis and genome maintenance | [77] | corpus luteum number |
| rs325939188 | *CLEC7A* | Sperm maturation and fertilization | [78] | reproductive tract weight |
| rs322659685 | *EML6* | Spermatogenesis | [79] | - |
| rs324930519 | *ABCB11* | Membrane transporter | [80] | - |
| rs343122214 | *SMS* | Protection of DNA from oxidative damage. | [81] | - |
| rs336346403 | *CDK17* | Sperm morphology | [82] | number of stillborn |
| rs334625232 | *ACSL4* | Phosphorylation and fatty acid metabolism | [83] | epididymis weight |
| rs328079913 | *ATP5F1A* | Motility and oxidative phosphorylation | [84] | - |
| rs335938037 | *HSPA13* | Sperm-oocyte interaction | [85] | - |
| rs81217594 | *PAM* | Biosynthesis of fatty acid primary amides | [86] | - |
| unknown | *RIOX2* | Ribosome biogenesis. Spermatogenesis | [87] | - |
| rs336351767 | *SKAP2* | Protein phosphorylation | [88] | - |
| rs326965968 | *MYO3B* | Spermatogenesis | [89] | litter weight |
| rs80954196 | *TMEM177* | Sperm-oocyte interaction | [90] | - |
| rs331568674 | *ROBO1* | Spermatogenesis | [91] | - |
| rs336003721 | *HARS2* | Aminoacylation and mitochondrial function. | [92] | litter size |

*Nearest genes found on the Ensembl database (http://www.ensembl.org/*Sus_scrofa*); GenBank accession nr: Acc
