# Supplementary material for: Gene Polymorphisms in Boar Spermatozoa and Their Associations with Post-Thaw Semen Quality
Source: Int J Mol Sci. 2020 Mar 10;21(5):1902. doi: 10.3390/ijms21051902 (PMC7084667; doi:10.3390/ijms21051902)
Supplement: Supplementary file 1 [file ijms-21-01902-s001.zip › Supplementary Table S5.docx]

**Supplementary Table S5**. Single nucleotide polymorphism (SNP) markers and primer sequences used for KASP genotyping assay.

| **SNP ID** | **Gene symbol** | **Primer sequences** | | | |
| --- | --- | --- | --- | --- | --- |
| unknown | *APPL1* | GAATCTGGAGGAGGT | [A/G] | TTAAATCCGAAACTG |  |
| rs338842672 | *CYP7B1* | TTTGCAGAGGCTGGA | [A/G] | GAGACGCTATTGAAC |  |
| rs345056502 | *EML5* | ATAAATTATATCATC | [A/G] | CCATCATTTAAATAA |  |
| unknown | *LPAR1* | AGTCCAGCAGATGAT | [A/G] | AAGGCGCCTGCAAGG |  |
| rs81210636 | *RAB3C* | AACTCAAATCAAAAC | [A/G] | TACTCATGGGATAAT |  |
| rs344846507 | *SARS* | AGTGTCTGCAGTAAG | [A/G] | TCATCAAGATTTAAT |  |
| rs340075321 | *TXNIP* | tttttttCCAATTCACTAA | [A/G] | GAACTGTAGTGGGAT |  |
| rs80886473 | *CRISP2* | TCCACATCCAACGCG | [A/G] | TAAGATGAGTACCAA |  |
| rs341011509 | *GLMN* | GCAGTTTAATATGGG | [A/G] | CACATTGAAGTCCTT |  |
| unknown | *IFNAR2* | AAAGATGAGATTCCG | [A/G] | AATTCCCAGTCAATC |  |
| rs196959943 | *AHI1* | GAACAAAATGTAGAT | [G/A] | ACAACATGCAAGAGG |  |
| rs339379734 | *OXSR1* | TCCAAGGTTGCAGCA | [G/A] | GATTAGTAAAAGGAA |  |
| rs339026428 | *A2M* | TGTACAGTAATGAAA | [G/A] | ATAGCCACAGGGTGG |  |
| rs81210697 | *ANKRD42* | TCGAAGATGTGGACT | [G/A] | CAATGGAAACCTTCC |  |
| rs332902509 | *CCDC149* | GAGGTTGACCTCTTC | [G/A] | TGAAGTTGCTTTAAT |  |
| unknown | *CFAP52* | CAACGTCTCCTGTGT | [G/A] | ACCATCTCCAAAAGT |  |
| rs318435440 | *COMMD2* | AGCACTGGGTGTTGC | [G/A] | TAGTAAAAAGATGGG |  |
| rs341614458 | *FBXO16* | ACCCTGCTCAAAGGG | [G/A] | GTTGGAGAGAAATTG |  |
| rs340643892 | *MAP3K20* | GATGAGTGTGTATGC | [G/A] | AGCTTGTTTAAGGAA |  |
| rs319208708 | *WRN* | AGTGCTGACTTTAGG | [G/A] | AGCCCAAAGTTCTGA |  |
| rs325939188 | *CLEC7A* | TTAGCACATCATTAG | [A/C] | TTCCTGGAATAGAAG |  |
| rs322659685 | *EML6* | CCATACCTTATCCAA | [A/C] | GCATACATAGCAAAC |  |
| rs324930519 | *ABCB11* | GCATGTGCCTACTGC | [C/A] | AGCTTAATTTCCTCTC |  |
| rs343122214 | *SMS* | TTTCACCCCCACCTC | [C/A] | CCTAAGACTCTTCTGT |  |
| rs336346403 | *CDK17* | TCTAATTAACCATGC | [C/A] | CCCAGGTAttttttttttCTCT |  |
| rs694366781 | *PROCKLE1* | ATAGGGAACCTTTTC | [C/T] | TCTGGTAAGCAAGCA |  |
| rs337913978 | *SLCT1* | TCATAAAGTCAGTGA | [C/T] | AAGGTGaaaaaaaaTATT |  |
| rs334625232 | *ACSL4* | CACACCAAGTTACTG | [T/C] | ACAGATATCAGTCCC |  |
| rs328079913 | *ATP5F1A* | TCTCAATTTGATAAG | [T/C] | TGCTTTCTAAAAASGG |  |
| rs325770408 | *CLNK* | GATCCCGGTTTTATC | [T/C] | TTCCCGTCAACGAGTA |  |
| rs335938037 | *HSPA13* | TCCTTGTTTATTCAG | [T/C] | AGAGAGACATCGAGA |  |
| rs81217594 | *PAM* | AGATTAAGCATGCAT | [T/C] | GTTCGGAAGAAATGT |  |
| unknown | *RIOX2* | GGCTTGTGTTCGATA | [T/C] | GGCAAAGGCAGATGT |  |
| rs336351767 | *SKAP2* | ATCGAACCACTCTTT | [T/C] | CCAAAGCGAAGTTTG |  |
| rs326965968 | *MYO3B* | ACAGCTGTGGCTGGT | [T/C] | CTGGAGGTAAGAGGC |  |
| rs80954196 | *TMEM17* | AGCCTCCCCTGGGTC | [C/G] | TGCCCAAGTGGACAC |  |
| rs339836492 | *MS4A2* | AAAAATCCAGAAATA | [G/C] | CAAACTGTCCAAAAG |  |
| rs331568674 | *ROBO1* | TGAAACttttttttttAAGAT | [A/T] | CGAGGAGGAAAGCTC |  |
| rs321497623 | *PLBD1* | TTTTTCTCAGAAACT | [T/A] | AGCTTCAAGGAGATG |  |
| rs336003721 | *HARS2* | CCTGATGCAGAATGT | [G/T] | TGAAGATCATGTGTG |  |
