# Supplementary material for: Gene Polymorphisms in Boar Spermatozoa and Their Associations with Post-Thaw Semen Quality
Source: Int J Mol Sci. 2020 Mar 10;21(5):1902. doi: 10.3390/ijms21051902 (PMC7084667; doi:10.3390/ijms21051902)
Supplement: Supplementary file 1 [file ijms-21-01902-s001.zip › Supplementary Table S2.docx]

**Supplementary Table S2**. KEGG pathway of enriched candidate genes (CGs) of boar spermatozoa.

| **ID** | **Term** | **GO Counts** | ***p-*value** | **CG name** |  |
| --- | --- | --- | --- | --- | --- |
| ssc05145 | Toxoplasmosis | 8 | 0.00107059374932 | *JAK1, IFNGR1, PIK3R1, JAK2, LAMA3, ITGB1, IRAK4, SLA-DRB1* |  |
| ssc05140 | Leishmaniasis | 7 | 0.00118958450208 | *JAK1, NCF4, IFNGR1, JAK2, ITGB1, IRAK4, SLA-DRB1* |  |
| ssc04724 | Glutamatergic synapse | 8 | 0.00236380661575 | *GLUL, SLC1A2, SLC1A1, SLC1A3, GRIA4, GNB5, ADCY2, GRM7* |  |
| ssc05162 | Measles | 8 | 0.00421592990494 | *CD28, JAK1, PIK3R1, SLAMF1, IFNGR1, JAK2, ENSSSCG00000009633, IRAK4* |  |
| ssc04550 | Signaling pathways regulating pluripotency of stem cells | 9 | 0.00502394525168 | *JAK1, WNT16, LHX5, ID4, PIK3R3, SMAD9, PIK3R1, JAK2, SMAD2* | |
| ssc04725 | Cholinergic synapse | 7 | 0.00646185289242 | *CAMK2D, PIK3R3, PIK3R1, GNB5, JAK2, ADCY2, CHRNA6* |  |
| ssc00120 | Primary bile acid biosynthesis | 3 | 0.00791896037004 | *CYP7A1, AMACR,* ***CYP7B1*** |  |
| ssc04151 | PI3K-Akt signaling pathway | 15 | 0.00800948308257 | *PKN1, FN1, JAK1, PIK3R3, PHLPP1, PIK3R1, YWHAH, IRS1, GNB5, JAK2, SGK3, PPP2R1B, ITGB1, ITGA2B, LAMA3* |  |
| ssc00270 | Cysteine and methionine metabolism | 5 | 0.00849869527317 | *BHMT2, AHCYL1,* ***SMS****, AGXT2, TST* |  |
| ssc00970 | Aminoacyl-tRNA biosynthesis | 5 | 0.00931922871593 | ***SARS****, TARS,* ***HARS2****, QRSL1, NARS* |  |
| ssc05222 | Small cell lung cancer | 6 | 0.00997541782558 | *FN1, PIK3R3, PIK3R1, LAMA3, ITGB1, ITGA2B* |  |
| ssc04923 | Regulation of lipolysis in adipocytes | 5 | 0.0121047040658 | *PIK3R3, CGA, IRS1, ADCY2, PIK3R1* |  |
| ssc04350 | TGF-beta signaling pathway | 6 | 0.0140867498946 | *RBL1, SMAD9, SP1, ID4, SMAD2, PPP2R1B* |  |
| ssc05164 | Influenza A | 9 | 0.0144983163752 | *VDAC1, JAK1, PIK3R3, IFNGR1, PIK3R1, JAK2, SLA-DRB1, ENSSSCG00000009633, IRAK4* |  |
| ssc04960 | Aldosterone-regulated sodium reabsorption | 4 | 0.0181499769769 | *PIK3R3, IRS1,ATP1A1, PIK3R1* |  |
| ssc05152 | Tuberculosis | 9 | 0.0187523170674 | *CEBPG, JAK1, CAMK2D,* ***CLEC7A****, NFYA, IFNGR1, JAK2, SLA-DRB1, IRAK4* |  |
| ssc05142 | Chagas disease | 7 | 0.0189895146466 | *IFNGR1, GNA14, PIK3R3, PIK3R1, SMAD2, PPP2R1B, IRAK4* |  |
| ssc04360 | Axon guidance | 9 | 0.020353225451 | *DPYSL2, SEMA6D, PIK3R3, CAMK2D, SRGAP1, PIK3R1,* ***ROBO1****, ITGB1, SEMA3E* |  |
| ssc05412 | Arrhythmogenic right ventricular cardiomyopathy (ARVC) | 5 | 0.0253155580277 | *DSC3, ITGB1, DSG2, GJA1, ITGA2B* |  |
| ssc04931 | Insulin resistance | 7 | 0.0255277091304 | *PYGB, MLXIPL, PIK3R3, MGEA5, PIK3R1, IRS1, COMMD6* |  |
| ssc04512 | ECM-receptor interaction | 5 | 0.0305752175014 | *ITGB1, FN1, ITGA2B, SV2B, LAMA3* |  |
| ssc05146 | Amoebiasis | 6 | 0.0331592712899 | *FN1, GNA14, SERPINB10, PIK3R3, PIK3R1, LAMA3* |  |
| ssc04727 | GABAergic synapse | 5 | 0.0344357833155 | *GABARAPL2, GABRR2, GNB5, ADCY2, GLUL* |  |
| ssc04974 | Protein digestion and absorption | 5 | 0.0430271874886 | *PRCP, ATP1A1, COL14A1, CPB1, SLC1A1* |  |
| ssc04022 | cGMP-PKG signaling pathway | 8 | 0.043755509184 | *VDAC1, PIK3R3, ATP1A1, PIK3R1, ATP2B1, ADCY2, IRS1, GTF2I* |  |
| ssc05033 | Nicotine addiction | 3 | 0.0475928394343 | *GABRR2, GRIA4, CHRNA6* |  |
| ssc01100 | Metabolic pathways | 38 | 0.04923219835 | *TST, AHCYL1, CYP7A1, GMPS, TRIT1,* ***ACSL4,*** *NMRK1****,*** *ALG11****,*** *GLUL****,*** *UAP1, AOC1, SDHC, ADH4, CPOX, LAMA3, NDUFS1, PRIM2,* ***SMS****, AASS, AGPS, GBA3, ASAH1, MTHFD1, AKR1C3, ATP5A1, MCCC2, PYGB, MGAT4A, AMACR, KMO, AGXT2, SDHB, HADHA, BHMT2, DMGDH, NMNAT1M, QRSL1, LIPG* |  |
